# GREATER DOPAMINERGIC SIGNALING IN PUTAMEN IS RELATED TO BETTER GAIT QUALITY IN ADULTS WITHOUT PARKINSON’S DISEASE

**DOI:** 10.1093/geroni/igae098.3662

**Published:** 2024-12-31

**Authors:** Mauricio Gil-Silva, Caterina Rosano, Nicolaas Bohnen, Stephen Kritchevsky, Eni Halilaj, Kayla Bohlke, Andrea Rosso

**Affiliations:** University of Pittsburgh, Pittsburgh, Pennsylvania, United States; University of Pittsburgh, Pittsburgh, Pennsylvania, United States; University of Michigan, Ann Arbor, Michigan, United States; Wake Forest University School of Medicine, Wake Forest University School of Medicine, North Carolina, United States; Carnegie Mellon University, Pittsburgh, Pennsylvania, United States; University of Pittsburgh Swanson School of Engineering, Pittsburgh, Pennsylvania, United States; University of Pittsburgh, Pittsburgh, Pennsylvania, United States

## Abstract

Gait quality aspects beyond pace like walking smoothness (harmonic ratio), regularity (entropy rate), and complexity (Lempel-Ziv complexity) may be useful in capturing the motor control strategies important in successful everyday walking. Dopaminergic signaling in the central nervous system plays a crucial role in motor function. Age-related declines in dopamine within the putamen may contribute to concurrent declines in gait quality in older adults without Parkinson’s. We investigated the relationship between putamen DA signaling and gait quality and hypothesized that lower DA signaling was associated with poorer gait quality. In 199 participants (Age: 75.00 (4.63), Sex: 123 F (61.81%) BMI: 28.22 (5.41)), (+)-[11C] (DTBZ) PET imaging estimated the striatal binding site density of the type 2 Vesicular Monoamine Transporter (VMAT2). Accelerometers on the L3 segment of the lumbar spine during walking over 15 meters measured entropy rate, Lempel-Ziv complexity, wavelet entropy, and harmonic ratio of the signal in the medial/lateral, anterior/posterior, and vertical anatomical directions. Multiple linear regression estimated the cross-sectional association between the posterior putamen and each gait measure, adjusted for age, sex, BMI, race, education, and hours walking per week. Lower posterior putamen DA binding site density was associated with both greater vertical wavelet entropy (-0.243 [-0.425, -0.060]) and greater medial/lateral Lempel-Ziv complexity (-0.045 [-0.083, -0.007]). Positive trending associations (p=< 0.10) for posterior putamen DA binding site density were found in medial/lateral entropy rate (0.072 [-0.010, 0.155]) and medial/lateral wavelet entropy (0.256 [-0.059, 0.571]). These results suggest that dynamic complex gait functions are more stable with higher putaminal DTBZ binding.

